# The Impact of Nitration on the Structure and Immunogenicity of the Major Birch Pollen Allergen Bet v 1.0101

**DOI:** 10.1371/journal.pone.0104520

**Published:** 2014-08-15

**Authors:** Chloé Ackaert, Stefan Kofler, Jutta Horejs-Hoeck, Nora Zulehner, Claudia Asam, Susanne von Grafenstein, Julian E. Fuchs, Peter Briza, Klaus R. Liedl, Barbara Bohle, Fátima Ferreira, Hans Brandstetter, Gertie J. Oostingh, Albert Duschl

**Affiliations:** 1 Department of Molecular Biology, University of Salzburg, Salzburg, Austria; 2 Department of Pathophysiology and Allergy Research and the Christian Doppler Laboratory for Immunomodulation, Medical University of Vienna, Vienna, Austria; 3 Christian Doppler Laboratory for Allergy Diagnosis and Therapy, Department of Molecular Biology, University of Salzburg, Salzburg, Austria; 4 Institute of General, Inorganic and Theoretical Chemistry/Theoretical Chemistry and Center for Molecular Biosciences Innsbruck (CMBI), University of Innsbruck, Innsbruck, Austria; Russian Academy of Sciences, Institute for Biological Instrumentation, Russian Federation

## Abstract

Allergy prevalence has increased in industrialized countries. One contributing factor could be pollution, which can cause nitration of allergens exogenously (in the air) or endogenously (in inflamed lung tissue). We investigated the impact of nitration on both the structural and immunological behavior of the major birch pollen allergen Bet v 1.0101 to determine whether nitration might be a factor in the increased incidence of allergy. Bet v 1.0101 was nitrated with tetranitromethane. Immune effects were assessed by measuring the proliferation of specific T-cell lines (TCLs) upon stimulation with different concentrations of nitrated and unmodified allergen, and by measurement of cytokine release of monocyte-derived dendritic cells (moDCs) and primary DCs (primDCs) stimulated with nitrated versus unmodified allergen. HPLC-MS, crystallography, gel electrophoresis, amino acid analysis, size exclusion chromatography and molecular dynamics simulation were performed to characterize structural changes after nitration of the allergen. The proliferation of specific TCLs was higher upon stimulation with the nitrated allergen in comparison to the unmodified allergen. An important structural consequence of nitration was oligomerization. Moreover, analysis of the crystal structure of nitrated Bet v 1.0101 showed that amino acid residue Y83, located in the hydrophobic cavity, was nitrated to 100%. Both moDCs and primDCs showed decreased production of T_H_1-priming cytokines, thus favoring a T_H_2 response. These results implicate that nitration of Bet v 1.0101 might be a contributing factor to the observed increase in birch pollen allergy, and emphasize the importance of protein modifications in understanding the molecular basis of allergenicity.

## Introduction

Over the past several decades, the incidence of allergy has increased in industrialized countries, affecting about 25% of the population [1]. Different factors have been blamed for this increase, including pollution [2], [3]. Air pollution is associated with enhanced levels of ozone, nitrogen dioxide, sulfur dioxide, and particulate matter. The complex relationship between the effects of air pollution and the occurrence of allergic diseases was reviewed in [4].

One of the consequences of air pollution is nitration of proteins, which can be mediated by NO_2_ and O_3_ at atmospheric concentrations [5]. Thus, tyrosine residues of proteins present in aerosol particles, such as pollen fragments, may be nitrated in polluted urban air before entering the airways. As shown by Franze et al., nitrated proteins can be found in urban road dust, window dust and air particulate matter, with degrees of nitration ranging from 0.01% to 0.1% [6].

In addition, pollution can also exacerbate airway inflammation, which leads to increased neutrophil, B-cell and alveolar macrophage recruitment [2], [7]. Airway inflammation has also been associated with increased NO levels, leading to greater formation of reactive nitrogen species and subsequent oxidation and nitration of proteins [8], [9]. Thus, pollution could also lead to nitration of allergens inside the lung tissue.

In Europe, birch pollen (BP) allergy is one of the most prevalent allergies, with Bet v 1.0101 being the major BP allergen. More than 95% of BP-allergic patients have specific IgE antibodies for Bet v 1.0101 [10]. Therefore, Bet v 1.0101 represents a suitable model to test the possible consequences of nitration. As described by Gruijthuijsen et al., higher levels of IgE antibodies against nitrated Bet v 1.0101 (termed nitro-Bet v 1.0101 hereafter) could be detected in comparison to unmodified Bet v 1.0101 in the blood of BP-allergic patients. Additionally, for nitro-Bet v 1.0101, an enhanced allergenic potential could be demonstrated in mice [11]. Recently, it was shown that nitration of Bet v 1.0101 increases the presentation of allergen-derived peptides by antigen-presenting cells (APCs), both quantitatively and qualitatively. Moreover, enhanced proliferation of Bet v 1.0101-specific T-cell lines (TCLs) occurred upon stimulation with nitro-Bet v 1.0101 in comparison to mock-nitrated Bet v 1.0101 (termed mock-Bet v 1.0101 hereafter) [12]. These findings indicate that nitration of Bet v 1.0101 might influence the immune response, although the molecular mechanisms behind this observation were not clear.

The physicochemical properties of tyrosine make it the amino acid that is most effective for mediating molecular recognition [13]. Because nitration of proteins mainly affects tyrosine residues, this change might lead to differences in recognition, resulting in altered protein activity [14].

To elucidate the possible mechanism for an altered immune response after nitration of Bet v 1.0101, we combined structural and immunological approaches. The structural approach encompasses three levels: the T-cell epitope level, the monomeric crystal structure of nitro-Bet v 1.0101, and the oligomerization state of nitro-Bet v 1.0101. The immunological set-up contains the two main players involved in initiation and polarization of immune responses: dendritic cells (DCs) and T cells.

We provide two possible explanations of the immunological effects based on structural alterations, seen after nitration of Bet v 1.0101, which could support the hypothesis that pollution can enhance the incidence of allergy.

## Materials and Methods

### Allergen preparation

Recombinant Bet v 1.0101 was expressed in *E. coli* strain BL21 (DE3) and purified as described elsewhere [15]. Endotoxin content was 40.5 ng/mg Bet v 1.0101 as determined by limulus amebocyte lysate (LAL) assay (Cape Cod, Falmouth, MA).

### Nitration of Bet v 1.0101 with tetranitromethane

Bet v 1.0101 was nitrated with tetranitromethane (TNM), diluted in MeOH, at a molar ratio of 30/1, 15/1, 5/1 and 1/1 TNM/tyrosine, leading to the formation of nitro-Bet v 1.0101 [16]. Simultaneously, a mock-control (MeOH only) was generated. After (mock-) nitration, the sample was centrifuged through a 10 kDa ultrafiltration tube (Merck Millipore, Cork, Ireland) to remove the reagents, and rinsed twice with fresh phosphate buffer.

### T-cell proliferation assay

Blood samples were analyzed in an anonymous manner after informed written consent was obtained from the allergic individuals with approval of the local ethics committee, Medical University of Vienna, Austria (EK number 028/2006). Allergen-specific TCLs were generated by stimulating peripheral blood mononuclear cells (1.5×10^6^) with 50 µg/ml BP extract, as previously described [17]. TCLs were stimulated with varying concentrations of Bet v 1.0101, mock-Bet v 1.0101 and nitro-Bet v 1.0101 (0.625–5 µg/ml), or unmodified and nitrated peptide. The peptides (piCHEM, Graz, Austria) analyzed were AA 4–18 (NYETETTSVIPAARL), AA 112–123 (SILKISNKYHTK) and AA 142–156 (TLLRAVESYLLAHSD). The stimulation index (SI) was calculated as the ratio between counts per minute (cpm) in stimulated cultures and cpm of the medium control.

### Biochemical characterization of the nitrated allergen

The concentration of the proteins and the degree of nitration were determined by amino acid analysis, and additionally the extent of nitration of the individual tyrosines was measured by high-performance liquid chromatography mass spectrometry (HPLC-MS). The oligomerization profile was investigated by SDS-PAGE, Western blot, and size exclusion chromatography (SEC). SEC was performed using a Superdex 75 10/300 GL column (GE Healthcare) with a running buffer of 10 mM phosphate buffer (salt free). The eluting protein was collected in 500 µl fractions. These fractions were analyzed by SDS-PAGE (15% SDS, non reducing). The whole samples were analyzed on a NuPAGE 4–12% Bis-Tris gel (Novex, Carlsbad, CA), visualized by Coomassie Brilliant Blue R-250 (Biorad, Hercules, CA) followed by Silver staining (Silver Quest, Invitrogen, Carlsbad, CA). As molecular weight marker, the SDS-PAGE Broad Range Standard (Bio-Rad, Hercules, CA) was used. Samples were analyzed by Western Blot, stained with an anti-nitrotyrosine rabbit antibody as primary antibody and a HRP-linked anti-rabbit IgG antibody as secondary antibody (both from Cell Signaling, Danvers, MA). As molecular weight marker, the Full Range Rainbow MW marker (GE Healthcare, Freiburg, Germany) was used. Additionally, dynamic light scattering (DLS) measurements were performed using a DynaPro Nanostar.(Wyatt Technology, Santa Barbara, CA, USA) using a 120 mW laser of 658 nm wavelength at 100% power. The samples were incubated at 25°C prior to the measurements. Two acquisition setups were used (20×10 s and 40×5 s) and were analyzed using the Dynamics 7.0.2 software (Wyatt Technology, Santa Barbara, CA, USA) with 10 mM PO_4_ as reference solvent.

### Endolysosomal degradation assay

Total microsomal fractions were isolated from the murine JAWS II DC cell line to perform *in vitro* endolysosomal degradation assays as previously described [18]. Assays were performed with 0.3 µg/µl of allergen and 0.5 µg/µl of isolated microsomal proteins in a final volume of 15 µl containing 100 mM citrate buffer pH 4.8 and 2 mM dithiothreitol. Reactions were conducted for 0 to 48 h at 37°C and stopped by heat denaturation. Samples were analyzed by SDS-PAGE, on a NuPAGE 4–12% Bis-Tris gel (Novex, Carlsbad, CA).

### Stimulation of monocyte-derived dendritic cells and cytokine profiling

Monocyte-derived dendritic cells (moDCs) were generated as previously described [19], with minor modifications. MoDCs were stimulated with 100 µg/ml Bet v 1.0101, mock-Bet v 1.0101 or nitro-Bet v 1.0101 nitrated to different degrees, and with 10 ng/ml lipopolysaccharide (LPS) (055:B5, Sigma-Aldrich, Vienna, Austria) as a control. Supernatant was taken after 24 hours and used for cytokine analysis (IL-12, TARC, TNF-α, IL-10 and IL-6) by means of ELISA (Peprotech, Rocky Hill, NJ).

### Stimulation of primary dendritic cells and cytokine analysis

Primary human CD1c^+^ dendritic cells (primDCs) were isolated using a MACS BDCA1+ kit (Miltenyi Biotech, Bergisch Gladbach, Germany), according to the manufacturer's instructions. Cells were stimulated with 50 µg/ml unmodified or nitrated protein, and with 10 ng/ml LPS as a control. Additionally, LPS-free Bet v 1.0101 and nitro-Bet v 1.0101 (50 µg/ml) were used for stimulation of primDCs. LPS removal from the allergen was performed with EndoTrap red (Hyglos, Regensburg, Germany) and assessed by LAL assay (0.8 ng/mg protein). Cytokine analysis was performed after 24 hours of stimulation by means of ELISA.

### Crystallisation of monomeric nitro-Bet v 1.0101

Nitro-Bet v 1.0101 was gel filtrated using a Superdex75 column (running buffer of 20 mM imidazole, pH 7.4, and 50 mM NaCl) to separate nitro-Bet v 1.0101 oligomers from monomers. Collected fractions containing exclusively monomeric nitro-Bet v 1.0101 were used for crystallisation. Crystallisation buffer was composed of 2.0 M ammonium sulfate as main precipitant and 1.5% MPD. Nitro-Bet v 1.0101 was used at 5 mg/ml protein concentration. Protein solution was mixed with crystallisation buffer in a 2∶1 ratio. After equilibration using sitting drop vapour diffusion, the drops were seeded with micro-seeds [20], obtained by wild type Bet v 1.0101 crystals, grown in similar conditions.

### Data collection and structure determination

Crystals were flash-frozen in a stream of nitrogen gas at 100 K. X-ray diffraction data sets were collected at beamline ID14-4 at the European Synchrotron Radiation Facility (ESRF, Grenoble). Diffraction data were indexed, scaled and further processed using CCP4 software suite [21]. The structure was solved by molecular replacement, using Phaser [22]. As search model, Bet v 1.0801 (PDB-ID 4A88) [23] was used. Refinement was performed using Refmac5 [24], and monitored throughout using an R_free_ calculated with 5% of the unique reflections. Model building was done in COOT [25]. All figures were generated using PyMOL [26]. Data collection and processing statistics are summarized in Table 1. Prior to deposition, the quality of the model was checked using MolProbity [27], NQ-Flipper [28] and PROCHECK [29]. Coordinates have been deposited with accession code 4B9R.

**Table 1:** X-ray data collection and model refinement statistics.

PDB-accession code	4B9R
**Data Collection**	
Spacegroup	P2_1_
X-ray source	Synchrotron
Cell Dimensions	
a, b, c (Å)	32.4/55.1/37.6
α, β, γ	90.0/92.7/90.0
Wavelength (Å)	0.97370
Number of unique reflections	12627
Resolution (Å)	37.57 - 1.76
R_merge_ (%)	0.060 (0.402)
Completeness (%)	95.9 (92.3)
Multiplicity	3.5 (3.3)
I/s(I)	12.7 (3.1)
Wilson B-factor	20.1
**Refinement Statistics**	
Resolution range (Å)	37.6 - 1.76
Number of unique reflections	11984
R_work_/R_free_ (%)	16.42/24.91
Number of Atoms	
Protein	1271
Water	104
Ligands	5
rmsd from ideal values	
bond lengths (Å)	0.007
bond angles (°)	1.281
Average B-factors (Å^2^)	23.7
Protein	22.9
solvent	33.8
nitro-Tyr	21.3

### Molecular Dynamics Simulations

We simulated three systems of native and nitrated Bet v 1.0101 (Table 2) to study the impact of nitration on the molecular dynamics using the simulation software AMBER11 [30] and the force field ff99SB [31]. Simulated systems were prepared from the X-ray structure of monomeric nitro-Bet v 1.0101 (PDB code: 4B9R) and native Bet v 1.0101 as published [23]. Nitration patterns of the simulated systems were chosen in respect to the experimental observations and are summarized below. Force field parameters for the nitro-tyrosine residues were extracted from the publication of Myung and Han [32]. Non-protein residues were removed from the starting structure. Hydrogen atoms were added and surface exposed histidine residues His76, His121, His154 were protonated and His126 was prepared as tautomer with hydrogen on the epsilon nitrogen (HIE). Thus, consistent protonation states for all systems were ensured. Additional to water molecules resolved in the crystal structures, a solvent box of around 8500 water molecules using TIP3P parameters was constructed with the Ambertool ‘leap’ for each system [30].

**Table 2:** Systems used for molecular dynamics simulations.

System	Native Bet v 1.0101	Denitro-Bet v 1.0101	Nitro-Bet v 1.0101
**Starting structure**	PDB code: 4A88	Adapted from 4B9R	PDB code: 4B9R
**Nitration pattern**	None	None	5, 66, 83, 150
**Number of atoms**	28090	28765	28725

Minimization and equilibration of the systems followed a multi-step protocol as previously described [33], including heating and annealing steps for water and protein equilibration with stepwise decrease of harmonic positional constraints using AMBER module ‘sander’. Productive simulations were performed on Nvidia GPUs with the AMBER module ‘pmemd.cuda’ using Particle Mesh Ewald summation with a cut off of 8 Å. Neutralizing plasma was applied to balance the total charge of −2 in the systems. Pressure was set constant at 1bar and temperature at 300 K controlled by Langevin thermostat. SHAKE algorithm fixing bond lengths to hydrogen atoms allowed a time step of 2 fs. Simulations were performed for 200 ns saving 2000 frames per ns to trajectories subsequently analyzed with ‘ptraj’ from Ambertool [30].

Stability and convergence of the trajectories were monitored as root-mean-square deviation (RMSD) of the C-alpha atom positions to the starting structure and 2-dimensional C-alpha RMSD between snapshots from each trajectory. The trajectories were analyzed in respect to residue-wise atomic fluctuations calculated as b-factor, secondary structure elements, hydrogen bond patterns and surface accessibility of selected residues to investigate the effect of nitration on the simulated systems. Cluster analysis was performed for frames collected each 0.5 ns using RMS distances for an average linkage algorithm with 5 clusters.

### Statistical analysis

Statistical evaluations were calculated with the Student *t*-test and one-way ANOVA followed by the Tukey's multiple comparison test. A value of *P<.05* was considered statistically significant.

## Results

### Low concentrations of nitro-Bet v 1.0101 elicit strong T-cell proliferation

**Figure 1:**
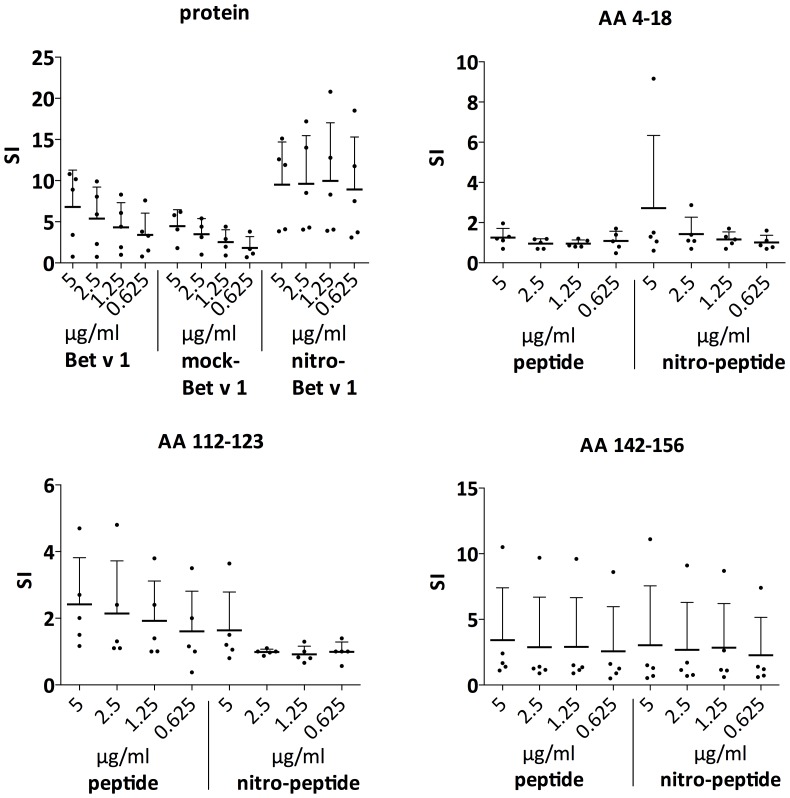
T-cell line proliferation. Proliferative responses of Birch pollen (BP)-specific TCLs from BP-allergic donors stimulated with decreasing amounts of LPS-contaminated Bet v 1.0101, mock-Bet v 1.0101 and nitro-Bet v 1.0101 (protein) and stimulated with decreasing amounts of three different LPS-free immunodominant epitopes (peptides) of Bet v 1.0101 containing a tyrosine, in native form or nitrated. The SI was calculated as the ratio between counts per minute (cpm) in stimulated cultures and cpm of the medium control. Each symbol represents a single donor (n = 5), the mean is indicated with a horizontal bar and the SD is represented by the vertical bar. Statistical significance was calculated with One-Way Anova and the Tukey's test per concentration (untreated, mock-nitrated and nitrated protein): *P* = .2506 for 5 µg/ml protein; *P* = .1354 for 2.5 µg/ml protein; *P = *.0815 for 1.25 µg/ml protein and *P* = .0618 for 0.625 µg/ml protein. Statistical significance was calculated with the *t*-test for the untreated and nitrated peptide per concentration. No values below *P* = .0824 could be measured.

As previously shown by our group, moDCs presented more Bet v 1.0101-specific peptides on MHC class II after 24 hours of stimulation with nitro-Bet v 1.0101 in comparison to mock-Bet v 1.0101. Additionally, Bet v 1.0101-specific TCLs showed stronger proliferation in response to nitro-Bet v 1.0101 compared to mock-Bet v 1.0101 [12]. In the present study, we compared the proliferation of BP-specific TCLs after stimulation with Bet v 1.0101, mock-Bet v 1.0101 and nitro-Bet v 1.0101, including lower protein concentrations than those used in the previous study (0.625–5 µg/ml). Nitro-Bet v 1.0101 caused a higher proliferation of BP-specific TCLs in comparison to Bet v 1.0101 or mock-Bet v 1.0101, which stayed high even at decreasing concentrations of allergen (Figure 1).

To determine whether this effect is due to the presence of nitro-tyrosine in the T-cell epitope presented, the cells were stimulated with three different peptides, representing the immunodominant epitopes of Bet v 1 [17], containing either a tyrosine or nitro-tyrosine. Only for one of the tested peptides (AA 4–18), one donor showed enhanced T-cell proliferation upon stimulation with the highest concentration of the nitrated peptide. For peptide 3 (AA 142–156), which was shown to be presented at a higher copy number by moDCs upon stimulation with nitro-Bet v 1.0101 in comparison to mock-Bet v 1.0101 [12], no difference in proliferation was observed after stimulation with unmodified or nitrated epitopes (Figure 1). Taken together, these data suggest that it is not the nitro group per se that contributes to the enhanced T-cell proliferation.

### Nitration of Bet v 1.0101 leads to oligomerization

**Figure 2:**
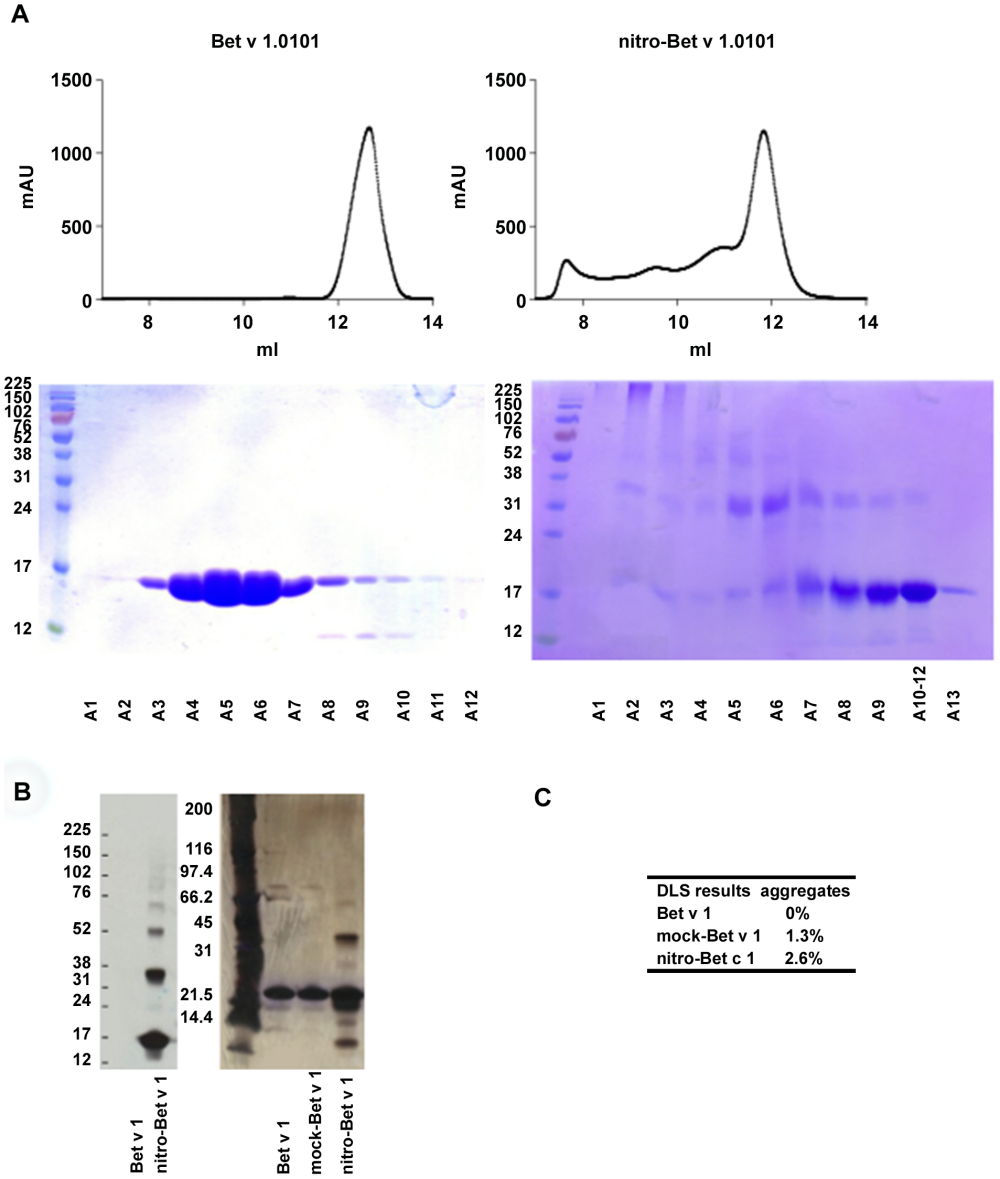
Biochemical characterization. Oligomerization and aggregation behavior of Bet v 1.0101, mock-Bet v 1.0101 and nitro-Bet v 1.0101 analyzed by size exclusion chromatography (SEC), by gel electrophoresis and by DLS. Highly concentrated sample (15 mg/ml) was loaded onto a Superdex 75 10/300 GL column for SEC analysis. Bet v 1.0101 and mock-Bet v 1.0101 eluted as a monomeric peak, whereas nitro-Bet v 1.0101 showed several peaks corresponding to oligomers. An SDS-PAGE (15%, non-reducing) with the corresponding fractions as eluted during the SEC is shown. Bet v 1.0101 was collected starting from an elution volume of 11 ml, nitro-Bet v 1.0101 was collected starting from an elution volume of 7 ml. *(A)* Nitro-tyrosine was detected with anti-nitrotyrosine antibody on Western blot and the whole protein was visualized by silver staining on SDS-PAGE. *(B)* DLS measurements revealed different aggregation states for Bet v 1.0101, mock-Bet v 1.0101 and nitro-Bet v 1.0101. *(C)*.

To analyze the biochemical characteristics of the nitrated protein, gel electrophoresis, size exclusion chromatography (SEC) and DLS measurements were performed. SEC analysis and the corresponding SDS-PAGE showed that the most prominent effect of nitration of Bet v 1.0101 was the formation of oligomers (Figure 2). Because the fractions of nitro-Bet v 1.0101 that eluted first were too large to be visible on the SDS-PAGE, DLS measurements were performed. DLS analysis showed no aggregation for untreated Bet v 1.0101, whereas both mock-Bet v 1.0101 and nitro-Bet v 1.0101 contained about 1.3% and 2.6% aggregates, respectively.

### Nitro-Bet v 1.0101 is less susceptible to endolysosomal degradation

**Figure 3:**
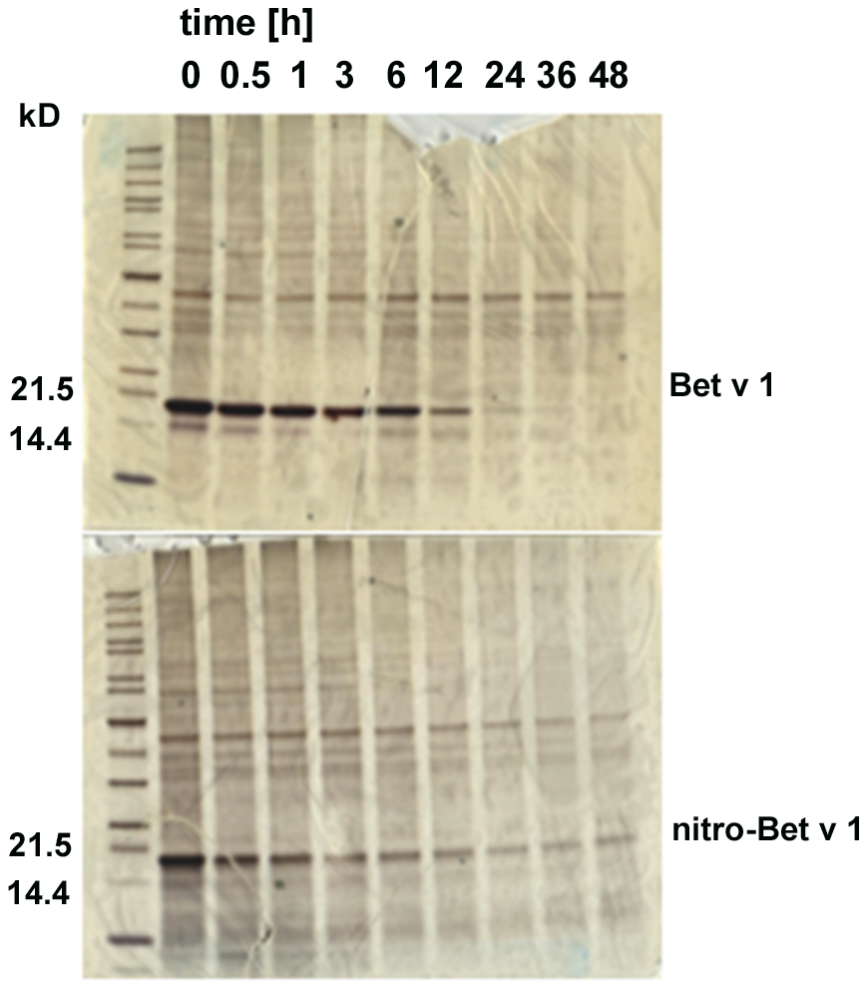
Endolysosomal degradation assay. Silver-stained SDS-PAGE of unmodified Bet v 1.0101 and nitro-Bet v 1.0101 after exposure to microsomal proteases isolated from a murine JAWS II DC cell line for different time points (0 to 48 hours), showing increased proteolytic resistance of nitro-Bet v 1.0101 in comparison to Bet v 1.0101.

As previously demonstrated, antigens less susceptible to lysosomal proteolysis show enhanced immunogenicity [34]. Both unmodified Bet v 1.0101 and nitro-Bet v 1.0101 were subjected to microsomal proteases and subsequently analyzed by SDS-PAGE (Figure 3). Degradation of Bet v 1.0101 occurred after 24 hours, whereas nitro-Bet v 1.0101 was still present after 48 hours, indicating increased proteolytic resistance of Bet v 1.0101 after nitration.

### Nitration of Bet v 1.0101 alters the cytokine secretion of DCs

**Figure 4:**
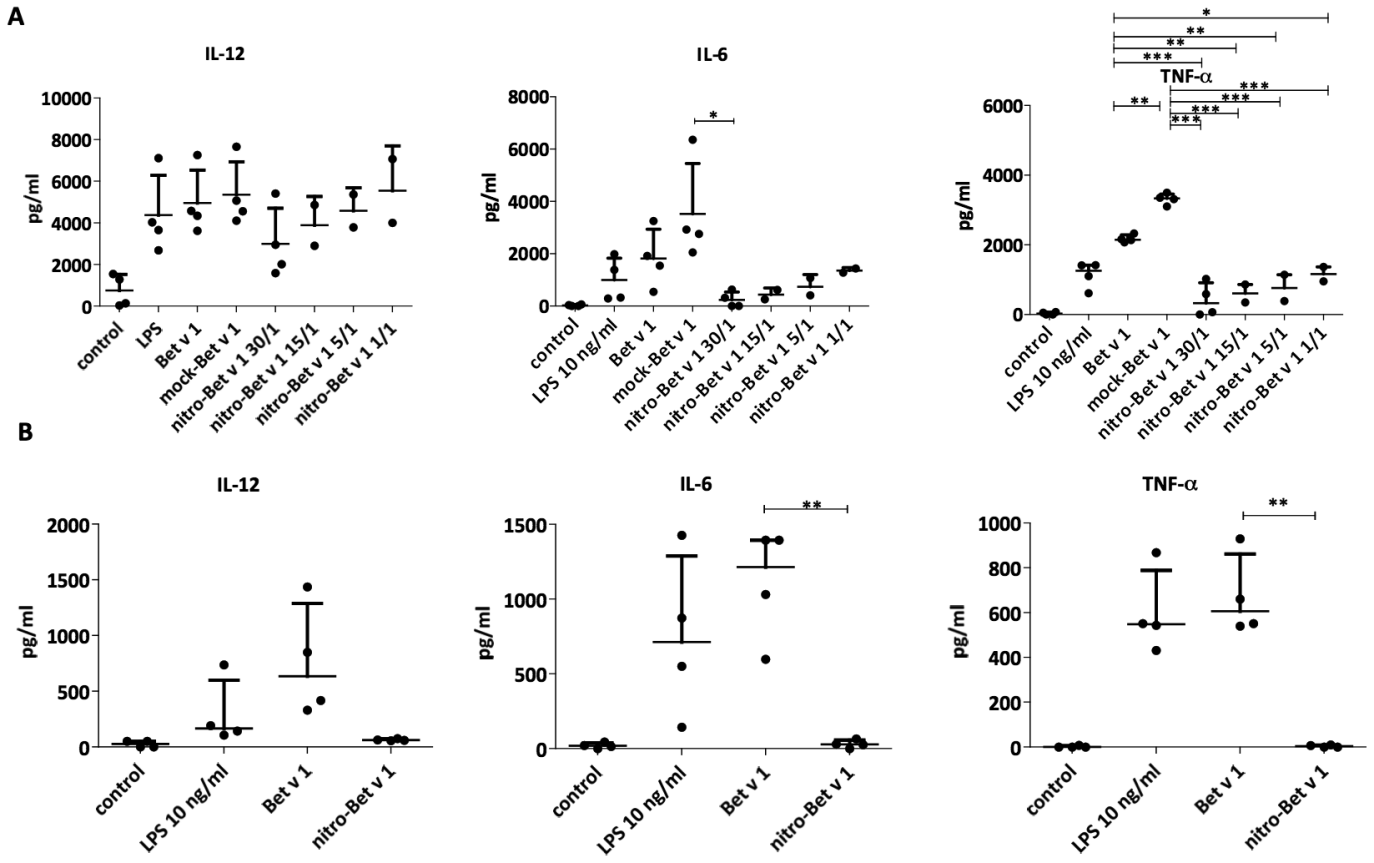
Cytokine profiles of DCs stimulated with LPS-contaminated allergen. Cytokine profiles of monocyte-derived DCs *(A)* and primary DCs *(B)* stimulated for 24 hours with LPS-contaminated Bet v 1.0101, mock-Bet v 1.0101 or nitro-Bet v 1.0101, nitrated to different degrees. MoDCs were stimulated with 100 µg/ml protein (total LPS contamination of 4 ng/ml) and primDCs with 50 µg/ml protein (total LPS contamination of 2 ng/ml). Each dot represents one donor. The mean and SD for all donors are shown. *P* values were calculated by one-way ANOVA (*P<.05; **P<.01; ***P<.001).

T cells recognize protein antigens as short peptides presented by APCs. However, the differentiation of T cells is determined by additional cytokines, secreted by APCs. Currently, the T_H_1-priming cytokines secreted by DCs are very well known, whereas those needed for induction of T_H_2-type immunity remain poorly understood, and the most prominent feature known is a lack of IL-12 secretion [35]. As shown by Eiwegger et al., LPS contamination in allergen preparations promotes Toll-like receptor 4 (TLR4) engagement [36]. We opted to include this engagement in order to obtain a measurable cytokine response, and thus in a first approach did not remove the LPS from the allergen preparation. The T_H_1-associated and pro-inflammatory cytokines IL-12, TNF-α and IL-6 were induced upon stimulation with Bet v 1.0101 and mock-Bet v 1.0101. However, secretion was significantly down-regulated upon stimulation with nitro-Bet v 1.0101 (Figure 4A). The same effect could be observed in primDCs (Figure 4B), which were used to confirm our findings under conditions more closely resembling the actual human immune response. Thus, nitration strongly inhibited the release of pro-inflammatory cytokines induced by LPS-contaminated Bet v 1.0101.

**Figure 5:**
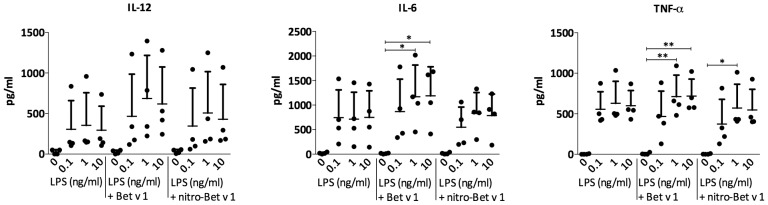
Cytokine profiles of primDCs stimulated with LPS-depleted allergen + LPS. Profile of cytokines released by primary dendritic cells after 24 hours of stimulation with different concentrations of LPS (0.1, 1 or 10 ng/ml) alone, or in combination with 50 µg/ml LPS-depleted Bet v 1.0101 or LPS-depleted nitro-Bet v 1.0101. Each dot represents one donor. The mean and SD for all donors are shown. *P* values were calculated by one-way ANOVA (*P<.05; **P<.01).

In a second approach, primDCs were stimulated with LPS-free Bet v 1.0101 or LPS-free nitro-Bet v 1.0101, alone or with increasing LPS concentrations (0.1, 1 or 10 ng/ml). No induction of IL-12, IL-6 and TNF-α was detected for the LPS-free stimuli. However, with the addition of external LPS, the latter cytokines were induced, but the strong inhibition of secretion of these cytokines previously seen for nitro-Bet v 1.0101 in comparison to Bet v 1.0101, was lost (Figure 5).

**Figure 6:**
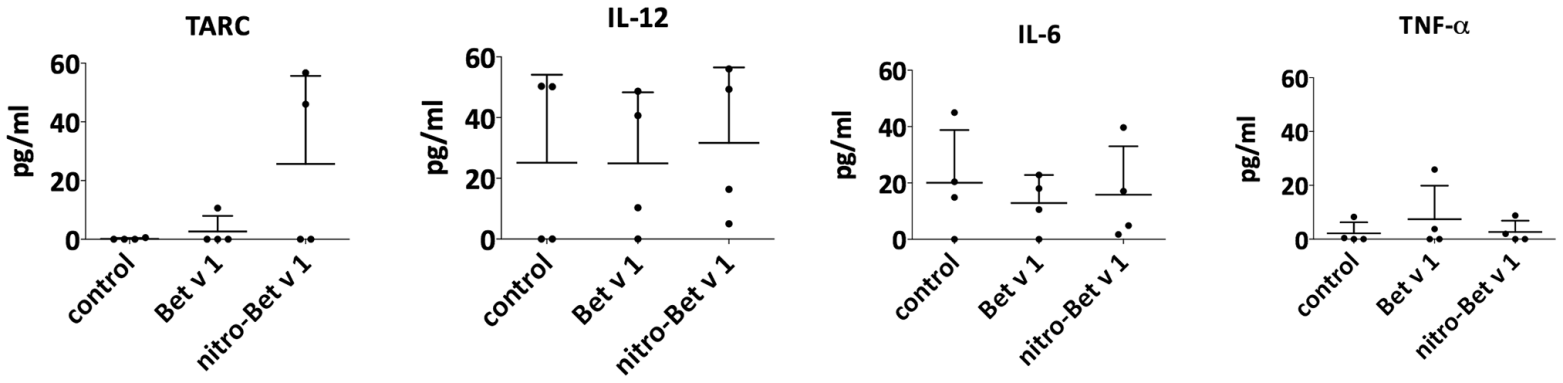
Cytokine profiles of primDCs stimulated with LPS-depleted allergen. TARC versus T_H_1-priming and pro-inflammatory cytokine secretion in primary DCs after 24 hours of stimulation with 50 µg/ml LPS-depleted Bet v 1.0101 or nitro-Bet v 1.0101. Each dot represents one donor. The mean and SD for all donors are shown. *P* values were calculated by one-way ANOVA. No *P*<.05 could be measured.

It is known that the balance between the T_H_1- and T_H_2-type immune responses determines the induction of allergy. One T_H_2-associated chemokine secreted by DCs is TARC (CCL17), which was up-regulated in two out of four donors after stimulation of primDCs with LPS-free nitro-Bet v 1.0101 compared to LPS-free Bet v 1.0101. For the cytokines IL-12, IL-6 and TNF-α, no difference could be observed (Figure 6).

### Structural characterization of nitro-Bet v 1.0101

**Figure 7:**
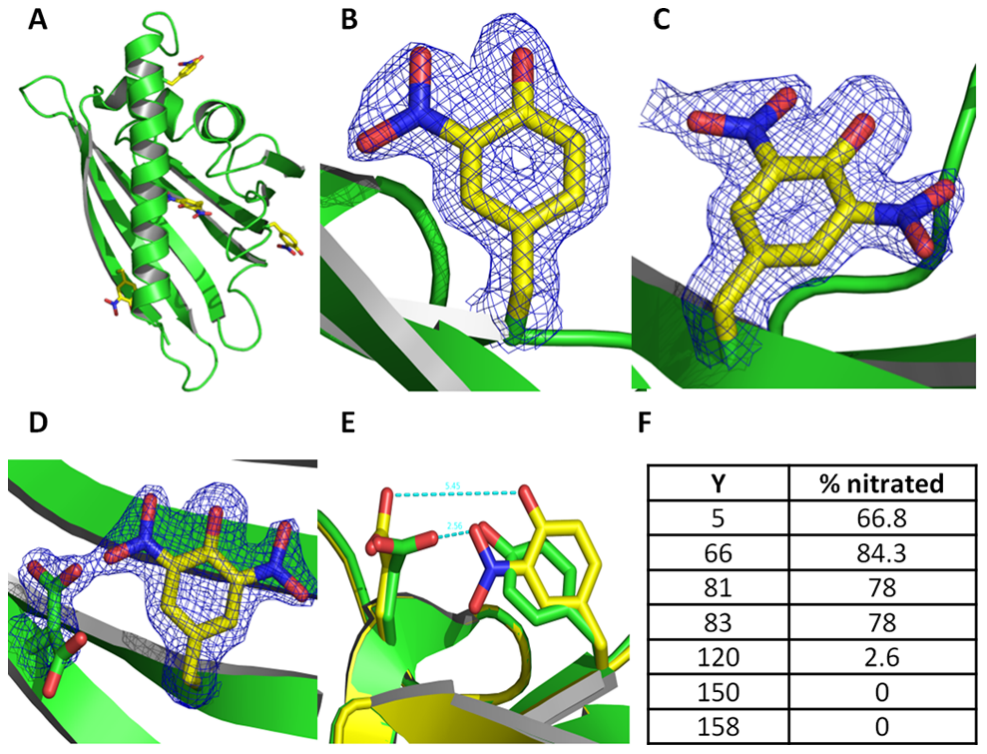
Structural analysis of nitro-Bet v 1.0101. Crystal structure analysis of monomeric nitro-Bet v 1.0101 revealed that monomeric nitro-Bet v 1.0101 displays the same backbone as Bet v 1.0101 with nitration of Y5, Y66, Y83 and Y150 *(A)*, the electron density corresponding to the nitration of tyrosine 66 *(B)*, the detection of two positions of the nitro group on tyrosine 83 *(C)*, the influence of nitration of Y83 on Asp 69 *(D)* and the influence of nitration of Y66 on Glu 87 *(E)*. HPLC-MS analysis of five separately nitrated Bet v 1.0101 samples for calculation of the mean percentage of nitration for each Y showed as main difference between the complete nitrated sample and the monomeric one the additional nitration of Y81 *(F)*.

To analyze the structure of monomeric nitro-Bet v 1.0101, and to gain knowledge of which tyrosines are nitrated, the crystal structure was solved to a resolution of 1.76 Å. It exhibits the classical Bet v 1.0101 fold and shows high similarity in comparison with the structure of wild-type Bet v 1.0101 crystallized under the same conditions (resolution 1.51 Å, pdb-accession code 4A88, [23]), resulting in an overall root-mean-square deviation (RMSD) of 0.23 Å. We could identify the electron density corresponding to a nitro group at four of seven tyrosine residues (Figure 7A): tyrosine 5, 66 and 150 (surface exposed) and 83 (located in the hydrophobic pocket). The occupancies of nitro groups vary strongly: 100% at both tyrosine (Y) 66 (Figure 7B) and Y83, 50% at Y5 and 20% at Y150. Tyrosine 83 in the hydrophobic core exhibits two conformations, described by a 180° rotation around the bond between Cγ and Cδ (Figure 7C). Compared to Bet v 1.0101, we found considerable changes in the side-chain orientation of both aspartic acid 69 (caused by nitro-tyrosine 83) (Figure 7D) and glutamic acid 87 (caused by nitro-tyrosine 66) (Figure 7E).

Although crystallization provides valuable information about the monomer, HPLC-MS analysis was used to analyze the nitration pattern of the whole nitrated sample, including the oligomers. A slightly different nitration pattern was revealed (Figure 7F), with the main difference being nitration of Y81 to the same extent as Y83.

### Molecular dynamics simulations

MD simulations were performed to study the effect of nitration on structure and dynamics in aqueous solution. Compared to the X-ray structure of Bet v 1.0101, the RMSD of the C-alpha atoms never exceeded 3.6 Å for all systems. Structural similarity of the systems is also reflected in similar residue-wise fluctuations in all systems. Generally, the investigated nitration pattern observed in the X-ray structure has no major effect on the overall dynamics of the Bet v 1 protein fold.

One of the local effects investigated in the MD simulations was solvent accessibility surface area (SASA) of tyrosine residues. We found accessibility of the side chain to be correlated with the presence of a nitro group in crystallized nitro-Bet v 1.0101 (Table 3). Consistently, all tyrosine residues with a surface above 50 Å^2^ were observed to be at least partially nitrated. The SASA of the side chains does not necessarily increase with the nitro group. Moreover, nitration has an effect on the neighboring tyrosine residues. Nitration at Y83 reduced the accessible surface of Y81 in comparison to simulations where Y83 was not nitrated. To a minor extent a similar effect was observed for Y158: if the neighboring Y150 is nitrated Y158 becomes less accessible.

**Table 3:** Solvent accessible surface area for tyrosine side chains.

	Y5*	Y66*	Y81	Y83*	Y120	Y150*	Y158
**Native Bet v 1.0101**	69.0 (23.2)	74.9 (16.6)	50.5 (10.5)	65.2 (11.2)	9.1 (11.7	71.6 (13.4)	38.8 (7.8)
**Denitro-Bet v 1.0101**	77.6 (24.8)	75.4 (16.9)	42.9 (8.2)	53.2 (9.3)	13.1 (14.5)	70.2 (13.2)	39.0 (8.0)
**Nitro-Bet v 1.0101**	64.8 (33.2)	92.7 (27.4)	24.1 (12.1)	55.2 (12.7)	38.1 (19.2)	73.5 (14.4)	30.8 (11.4)

Solvent accessible surface area (SASA) for tyrosine side chains calculated from MD simulations. Tyrosine residues with high solvent accessibility were nitrated in the X-ray structure (highlighted with *). In nitro-Bet v 1.0101 the SASA is decreased for Y81 and Y158, related to nitration of the proximate Y83* and Y150*, respectively. Numbers in brackets indicate standard deviations.

## Discussion

In this study, we looked for molecular findings that test the hypothesis that pollution potentially enhances the incidence of allergy. As reported by Karle et al., nitration of Bet v 1.0101 led to a qualitatively and quantitatively different presentation of MHC-associated peptides in comparison to mock-Bet v 1.0101 [12]. We showed here that BP-specific TCLs from allergic donors proliferate more strongly upon stimulation with nitro-Bet v 1.0101 in comparison to Bet v 1.0101 or mock-Bet v 1.0101. Moreover, the proliferation of T cells stayed high even at lower concentrations of nitrated allergen, in contrast to the comparatively low levels of proliferation induced by treatment with unmodified or mock-nitrated allergen. This indicates that, especially at low concentrations of allergen, nitro-Bet v 1.0101 shows strongly enhanced T-cell stimulatory capacities. Because this effect was not observed when the TCLs were stimulated with either nitrated or unmodified immunodominant epitopes of Bet v 1.0101, we hypothesize that it is not the nitro-group per se that drives the process, but rather that nitration might have an influence on the overall structure of Bet v 1.0101.

To analyze the overall structure, biochemical characterization of nitro-Bet v 1.0101 was carried out. The major biochemical effect of nitration is oligomerization, which was suggested to be caused through formation of dityrosine cross-links [37], [38]. It has been a matter of debate whether oligomerization leads to higher allergenicity or higher immunogenicity [39]–[42]. One hypothesis is that oligomers would resist endolysosomal degradation for a longer time, leading to extended presentation of the molecule. Indeed, we were able to observe this effect upon exposure of the nitrated versus unmodified allergen to endolysosomal proteases.

Subsequently, we investigated cytokine secretion by DCs. In a first approach, no endotoxin removal was conducted after expression of the allergen in *E. coli*. The secretion of IL-12, IL-6 and TNF-α by moDCs, induced by LPS-contaminated Bet v 1.0101, was significantly lower upon nitration, and was shown to depend on the degree of nitration. Notably, mock-Bet v 1.0101 induced an even stronger cytokine release compared to untreated Bet v 1.0101. As measured with DLS, both mock-Bet v 1.0101 and nitro-Bet v 1.0101 contain a small percentage of aggregates, probably induced by mechanical stress occurring during the (mock-) nitration reaction [43], [44]. As even small amounts of aggregates can induce high immunogenic responses [45], this might explain the effects seen with mock-Bet v 1.0101; however, they are counter-regulated by nitration. Therefore, we conclude that mock-Bet v 1.0101 may not be a suitable control and the effects of nitration should always be compared to untreated Bet v 1.0101 as well.

The induction of primDCs with LPS-free allergen was performed to refine the *in vitro* system. With LPS-free allergen alone, no difference in the release of T_H_1-priming cytokines could be measured, whereas the T_H_2-associated chemokine TARC (CCL17) was slightly up-regulated by nitro-Bet v 1.0101. Thus, we compared both LPS-free and LPS-contaminated allergen, left unmodified or nitrated, alone or in combination with additionally added LPS. This led to the interesting result that, although cytokine release was also diminished in DCs treated with nitrated LPS-free Bet v 1.0101 and externally added LPS in comparison to Bet v 1.0101, the most striking effect was observed for DCs stimulated with nitrated, LPS-contaminated Bet v 1.0101. Additionally, stimulation of DCs with nitrated LPS alone did not impact the cytokine secretion in comparison to stimulation with unmodified LPS (data not shown). These data together suggest a direct interaction of the protein and LPS, of which the synergistic effect could explain the decreased secretion of T_H_1-associated cytokines by DCs after stimulation with nitrated LPS-contaminated Bet v 1.0101.

A remarkable feature of Bet v 1.0101 is its large hydrophobic cavity, for which the natural ligand has not yet been found. As shown by Kofler et al., Y83 is involved in ligand binding of Bet v 1.0101 [23]. Thus, nitration at this position might have a detectable influence on ligand binding. X-ray analysis revealed that, under the same crystallisation conditions and after seeding with Bet v 1.0101, nitro-Bet v 1.0101 has a classical Bet v 1 fold. In MD simulations, no major differences in the dynamics of nitro-Bet v 1.0101 (based on X-ray structure) in comparison to the native protein could be observed. Four of 7 tyrosines were found to be nitrated, with different occupancies: 100% at Y66 and Y83, 50% at Y5 and 20% at Y150. Notably, Y83, which is located in the hydrophobic cavity of the protein, could be found in two conformations, one of which is responsible for a change in the side-chain orientation of aspartic acid 69. This allows for the speculation that, due to the nitration of Y83, the binding of a potential ligand can be weakened. As the most striking effects of nitration were observed for Bet v 1.0101 that was contaminated with LPS at low concentrations, one might speculate that LPS might serve as possible ligand of Bet v 1.0101. Thus, nitration of Y83, which is strongly involved in ligand binding of Bet v 1.0101, might result in a significant reduction of simultaneous TLR4 activation. Considering the T_H_1/T_H_2-balance that exists under normal conditions, absence of T_H_1 stimulation may lead to enhanced T_H_2 activation, which provides a molecular explanation for the higher allergenicity of nitro-Bet v 1.0101. Nitration of LPS itself was also investigated and did not show a similar effect. However, the increased T-cell proliferation detected earlier [12], was found in experiments performed with LPS-depleted allergen, thus excluding an explanation solely provided by LPS-modification.

A second explanation for the observed effects would be that nitration induces oligomerization, which is responsible for the stronger immune response. This assumption is substantiated by the fact that Y5, Y66 and Y150 are solvent exposed, and thus could undergo nitration and potentially form dityrosine cross-links with adjacent molecules, leading to oligomers. This might result in enhanced immunogenicity, as shown by decreased susceptibility toward endolysosomal degradation and increased T-cell proliferation. Interestingly, we can conclude from our study that aggregation does not provoke the same immunologic effects as oligomerization, as the effects seen for mock-Bet v 1.0101 are opposite to those seen for nitro-Bet v 1.0101. This second hypothesis is strengthened by the recent publication depicting a favored T_H_2-response in primDCs stimulated with dimeric Bet v 1.0101 in comparison to monomeric Bet v 1.0101 [46].

## Conclusion

In the present study we observed that nitration of the major BP-allergen alters both the biochemical and the immunological properties of the allergen. The main structural consequence of nitration of Bet v 1.0101 is oligomerization as well as nitration of the tyrosine residue involved in ligand binding of Bet v 1.0101. The main immunological impact of nitration of Bet v 1.0101 is an enhanced proliferation of BP-specific TCLs, a prolonged resistance towards endolysosomal degradation and a decreased secretion of T_H_1- and pro-inflammatory cytokines by DCs. The following model explaining the molecular mechanisms underlying enhanced allergenicity of nitro-Bet v 1.0101 can be proposed. Nitration of Bet v 1.0101 induces oligomerization, which consequentially increases the immunogenicity of the allergen. In addition, nitration may interfere with the ability of hydrophobic ligands (possibly LPS) to bind to the hydrophobic cavity of Bet v 1.0101. As a consequence, less LPS would be directly associated with nitro-Bet v 1.0101 compared to unmodified Bet v 1.0101, resulting in reduced secretion of T_H_1-priming cytokines needed to counter-balance T_H_2 inflammation. Thus, the enhanced immunogenicity of nitrated Bet v 1.0101 is shifted toward an increase in allergenicity.
